# Preclinical Development of Ipilimumab and Nivolumab Combination Immunotherapy: Mouse Tumor Models, In Vitro Functional Studies, and Cynomolgus Macaque Toxicology

**DOI:** 10.1371/journal.pone.0161779

**Published:** 2016-09-09

**Authors:** Mark J. Selby, John J. Engelhardt, Robert J. Johnston, Li-Sheng Lu, Minhua Han, Kent Thudium, Dapeng Yao, Michael Quigley, Jose Valle, Changyu Wang, Bing Chen, Pina M. Cardarelli, Diann Blanset, Alan J. Korman

**Affiliations:** Bristol-Myers Squibb, Redwood City, CA, United States of America; University of South Alabama Mitchell Cancer Institute, UNITED STATES

## Abstract

The monoclonal antibodies ipilimumab (anti-CTLA-4) and nivolumab (anti-PD-1) have shown remarkable antitumor activity in an increasing number of cancers. When combined, ipilimumab and nivolumab have demonstrated superior activity in patients with metastatic melanoma (CHECKMATE-067). Here we describe the preclinical development strategy that predicted these clinical results. Synergistic antitumor activity in mouse MC38 and CT26 colorectal tumor models was observed with concurrent, but not sequential CTLA-4 and PD-1 blockade. Significant antitumor activity was maintained using a fixed dose of anti-CTLA-4 antibody with decreasing doses of anti-PD-1 antibody in the MC38 model. Immunohistochemical and flow cytometric analyses confirmed that CD3^+^ T cells accumulated at the tumor margin and infiltrated the tumor mass in response to the combination therapy, resulting in favorable effector and regulatory T-cell ratios, increased pro-inflammatory cytokine secretion, and activation of tumor-specific T cells. Similarly, *in vitro* studies with combined ipilimumab and nivolumab showed enhanced cytokine secretion in superantigen stimulation of human peripheral blood lymphocytes and in mixed lymphocyte response assays. In a cynomolgus macaque toxicology study, dose-dependent immune-related gastrointestinal inflammation was observed with the combination therapy; this response had not been observed in previous single agent cynomolgus studies. Together, these *in vitro* assays and *in vivo* models comprise a preclinical strategy for the identification and development of highly effective antitumor combination immunotherapies.

## Introduction

Cytotoxic T-lymphocyte-associated protein 4 (CTLA-4) and programmed cell death protein 1 (PD-1), among other inhibitory T-cell surface molecules, attenuate a variety of activated T-cell functions, including cellular proliferation, cytokine secretion, and cytolysis [1]. Importantly, in the context of oncologic diseases, it has been demonstrated that tumor cells, as well as tumor-infiltrating host cells, express ligands for these inhibitory receptors that permit evasion of immunosurveillance [2]. Antibody blockade of CTLA-4 and PD-1 has resulted in dramatic reductions in tumor burden in many human subjects [3–5].

CTLA-4 has been shown to inhibit T-cell responses by both intrinsic and extrinsic mechanisms [6–10]. With respect to the intrinsic mechanism, engagement of CTLA-4 on T cells by B7 ligands leads to their functional attenuation. There are multiple extrinsic mechanisms that include the ability of CTLA-4-expressing cells to effectively compete with CD28 for B7 ligands or trans-endocytic removal of costimulatory ligands from antigen-presenting cells (APC) [11]. CTLA-4 is critical for the function of regulatory T cells (Tregs), which are essential for suppressing autoimmunity and for maintaining self-tolerance. Blocking antibodies to CTLA-4 have induced antitumor activity in syngeneic mouse tumor models [12]. Treatment of tumor-bearing mice with anti-CTLA-4 antibodies capable of depletion have been shown to substantially reduce Tregs in tumors but not in the periphery, resulting in potentiated antitumor activity as compared to antibodies that lack effector function [13–15].

PD-1 is an additional key inhibitory receptor with immunoreceptor tyrosine-based inhibitory motif (ITIM) and immunoreceptor tyrosine-based switch motif (ITSM) intracellular signaling motifs that strongly dampen T cell functions following engagement by its ligands PD-L1 and PD-L2 [16, 17]. PD-1 plays a central role in maintaining T cell tolerance [18]. Persistent high-level PD-1 expression on T cells is a part of a signature for non-responsive exhausted T cells associated with chronic lymphocytic choriomeningitis virus (LCMV) and human immunodeficiency virus (HIV) [19] as well as with tumors [20]. Ligand-blocking anti-PD-1 and anti-PD-L1 antibodies have shown antitumor activity in different models, demonstrating the role this pathway plays in limiting host antitumor responses.

Here we present the rationale and course of preclinical development of anti-CTLA-4 and anti-PD-1 combination immunotherapy. We first assessed whether the combination of mouse surrogate antagonist antibodies to these receptors could promote greater activity in preclinical tumor models both responsive and refractory to the individual therapies. As expression of CTLA-4 and PD-1 appear individually and jointly on multiple T cell subsets with differing levels and kinetics of expression, how these molecules interact to mediate suppression is not completely understood. We investigated different dosing regimens as well as the tumor microenvironment for changes in immune cell subsets and cytokine production as potential indicators of enhanced antitumor response. These data prompted evaluation of human antibodies ipilimumab and nivolumab in *in vitro* assays with human blood cells. Specifically, superantigen staphylococcal enterotoxin B (SEB) and mixed lymphocyte response (MLR) assays were conducted to compare human T cell immune responses in combination therapy and monotherapy. Subsequently, we assessed concurrent dosing of ipilimumab and nivolumab in a non-human primate study for potential immune-related adverse events (AEs). The results and utility of these preclinical experiments were validated by the observed superior clinical efficacy of combined ipilimumab and nivolumab in a phase 3 trial in patients with metastatic melanoma [21].

## Materials and Methods

### Antibodies and Tumor Challenge Experiments

The chimeric anti-mouse PD-1 antibody (4H2) used in this study, engineered as a mouse IgG1 isotype monoclonal antibody (mAb) [22] was shown to bind to CHO transfectants expressing PD-1 and block binding of PD-L1 and PD-L2 to these cells (S1 Fig). The affinity of 4H2 for mouse PD-1, determined by surface plasmon resonance using PD-1-Fc, was 4.68×10^−9^ M. The mIgG2b anti-mouse CTLA-4 mAb (9D9) used in this study was described previously [13]. All mAbs were formulated in sterile PBS and were low in endotoxin (<0.05 EU/mg).

MC38 tumor cells were cultured in DMEM and implanted subcutaneously into female C57/Bl6 from Charles River Laboratories (CRL) or B6.129S7-*Ifng*^*tm1Ts*^/J C57BL/6 from Jackson Laboratory (JAX) mice. CT26 tumor cells were cultured in DMEM and implanted subcutaneously in female BALB/c mice from CRL or Harlan Laboratories (HAR). Tumor measurements were made 2–3 times weekly using an electronic caliper (reported as either mm^3^ [L×W×H] or mm^3^/2). Except where indicated, antibody doses were administered intraperitoneally (IP) in total volumes of 200 μL on days 7, 10, and 13. For T-cell depletion studies, 500 μg of depleting antibodies for CD4 (GK1.5) or CD8 (53.6.72; BioXCell, W. Lebanon, NH) were administered on day 7 following subcutaneous implantation of MC38 tumor cells (2×10^6^) in the hind flank. The efficiency of CD4^+^ or CD8^+^ T cell depletion (>90%) was confirmed by FACS analysis of blood samples collected four days after administration of the depleting antibodies. Mice were sacrificed with CO_2_ at either study termination or any of the following clinical endpoints: tumor volume ≥2000 mm^3^, tumor ulceration, body weight loss ≥20%, or moribund appearance.

### Immune Response Monitoring

Tumors were harvested and processed using GentleMacs cell disruptors (Miltenyi). Resulting cell suspensions were clarified through 70 μM filters, pelleted, resuspended in PBS or DMEM, and counted. Cells were incubated with anti-CD16/32 mAb 24G.2 (BioXCell or BD Biosciences) to reduce background FcγR binding and then stained with antibodies specific for CD8 (BioLegend 53–6.7), CD4 (BioLegend GK1.5), and CD45 (BioLegend 30-F11). Cells were also stained with the LiveDead Aqua fixable viability dye (ThermoFisher L34597). For intracellular staining (ICS), samples were fixed, permeabilized, and stained with antibodies specific for FoxP3 (eBioscience FJK-16s), Ki67 (eBioscience SolA15), CTLA-4 (BD Pharmingen 4F10), IFN-γ (eBioscience XMG1.2), and TNF-α (MP6-XT22). CT26 tumor antigen-specific CD8^+^ T cells were identified using AH-1 MHC class I tetramers (MBL MuLV gp70 SPSYVYHQF). Tumor and splenic cell suspensions were incubated with tetramer in DMEM and 10% FCS for 30 minutes at 37°C, washed, and stained with surface and intracellular antibodies as above. *Ex vivo* AH-1 peptide stimulation was performed by culturing tumor or splenic cells with 2 μM AH-1 peptide (MBL) in the presence of brefeldin-A for 4 hours at 37°C. *Ex vivo* cytokine staining was performed by fixing and staining cells as described above, directly after tissue harvest. Samples were analyzed on FACS Canto and Fortessa flow cytometers (BD Biosciences).

### Cytokine Assays of Tumor Harvests

Tumors were harvested into 1 mL of complete T-cell media in 24-well microtiter plates and manually dissociated into single-cell suspensions. Tumor homogenates were clarified by centrifugation and resulting supernatants were collected and frozen for later batch sample processing. A volume of 25 μL of neat supernatant from each sample was analyzed in duplicate for concentrations of intratumoral IL-1α, IL-2, IL-4, IL-5, IL-6, IL-10, IL-13, IL-17A, IL-21, IL-22, IL-27, IP-10, GM-CSF, TNF-α, and IFN-γ using a bead-based cytokine array according to the manufacturer’s instructions (FlowCytomix, eBioscience).

### Immunohistochemistry

MC38 tumors were embedded in optimal cutting temperature (OCT) compound, snap-frozen on dry ice, and stored at -80°C until staining. Serial 5 μm tumor sections were placed onto glass slides, dried overnight at room temperature (RT), and then stained or stored at -80°C until use. For immunohistochemical staining, air-dried slides were fixed with acetone at RT, treated with peroxidase block (Abcam) to quench endogenous peroxidase, and then further blocked with a 10% goat serum and 5% BSA solution. Sections were stained with rabbit anti-mouse CD3 antibody (Abcam SP7) at a 1:400 dilution or rat anti-mouse PD-L1 (eBioscience MIH5) at a 1:100 dilution in blocking buffer. CD3^+^ T cells were detected using the EXPOSE rabbit-specific HRP-DAB detection kit (Abcam ab80437). PD-L1^+^ cells were detected using goat anti-rat-HRP (1:1000; Jackson ImmunoResearch 112-036-071) and detected with the HRP-DAB detection kit. Slides were co-stained with pre-made hematoxylin (Biocare CATHE-M) to counterstain cell nuclei. A similar protocol was applied for staining of PD-L1.

### *In Vitro* Assays Using Human Lymphocytes

Frozen peripheral blood mononuclear cells (PBMC) from normal healthy leukophoresis donors were seeded at 1×10^5^ cells/well and stimulated with SEB serially diluted 30-fold from 2.5 μg/mL. Anti-CTLA-4 (ipilimumab), anti-PD-1 (nivolumab), or huIgG4 isotype control (Bristol-Myers Squibb 1D12-g4) was present at a spike concentration of 10 μg/mL. IL-2 secretion was measured by ELISA (BD Biosciences) on day 3.

Mixed lymphocyte response assays were performed by co-culturing 1×10^5^ cells CD4^+^ T cells with allogeneic monocyte-derived dendritic cells (DC) at a ratio of 10:1 (T:DC) in flat-bottom 96-well microtiter plates. CD4^+^ T cells and DC were incubated for 6 days in the presence or absence of nivolumab titrated 1:10 from 50 mg/mL to 5 ng/mL along with ipilimumab at 0, 5, or 50 μg/mL. Culture supernatants were harvested on day 5 for ELISA analysis of IFN-γ secretion.

To assess the potential of nivolumab or ipilimumab, alone or in combination, to induce nonspecific T cell activation, mAbs were mixed with samples of heparinized fresh human whole blood to measure cytokine release. After a 4-hour incubation at 37°C, the cells were pelleted and the plasma fraction collected for measurement of IFN-γ, TNF-α, IL-2, IL-4, IL-6, and IL-10 using a cytokine cytometric bead array assay (BD Biosciences). Comparison was made to responses generated by anti-CD3 or isotype control mAb treatment.

### Cynomolgus Monkey Toxicity Study

A 4-week study was conducted in cynomolgus monkeys to evaluate the toxicity of co-administered ipilimumab and nivolumab. The study was conducted at CRL, Sparks, NV in compliance with the Good Laboratory Practice Regulations for nonclinical Laboratory Studies of the US Food and Drug Administration (21 CFR Part 58), the USDA Animal Welfare Act (9 CFR, Parts 1, 2, 3), and the Guide for the Care and Use of Laboratory Animals of the National Institutes of Health (ILAR publication 1996). The study-specific protocol was approved by the CRL Institutional Animal Care and Use Committee (IACUC). In addition to twice daily cage side observations by CRL staff, weekly physical examinations were performed by a CRL veterinarian.

Thirty purpose-bred cynomolgus monkeys (5/sex/group) were assigned to 3 groups by a stratified randomization scheme designed to achieve similar group mean body weights and the groups were randomly assigned to treatment. The groups were dosed intravenously (IV) with 1) saline control, 2) nivolumab 10 mg/kg plus ipilimumab 3 mg/kg, or 3) nivolumab 50 mg/kg plus ipilimumab 10 mg/kg, once weekly (days 1, 8, 15, and 22), for a total of 4 doses. The animals were housed individually, but were commingled daily to provide for environmental enrichment. Animals were fed Purina Certified Primate Diet No. 5048, and fruits, vegetables, and other treats were provided for environmental enrichment. The animals were evaluated for changes in clinical signs daily and body weights were recorded weekly. Cardiovascular assessments were performed prior to study and on days 1 and 22. Clinical pathology assessments were performed prior to study and on days 7, 28, and 58.

Seventeen monkeys (3/sex/group for Groups 1 and 2, and 2 males/3 females for Group 3) were euthanized 8 days after the last mAb dose, and 12 monkeys (2/sex/group) were euthanized on day 59. Animals were euthanized by exsanguination while under deep anesthesia induced with ketamine and Beuthanasia-D or equivalent. A full necropsy was conducted on all animals, with organs weighed and tissue collected, preserved, and processed for microscopic and histologic evaluations.

### Statistics

Statistical analyses of two groups were conducted using unpaired or paired two-tailed Student’s t-tests. Analyses of more than two groups were conducted using Kruskal-Wallis ANOVA with Dunn’s method of multiple comparisons against the control condition. Analyses of tumor growth measurements were performed by comparing tumor volumes measured on the last day on which all study animals were alive. * p<0.05; ** p<0.01; *** p<0.001. For each experiment, the number of replicates performed and the number of animals per group are described in the corresponding figure legend(s).

## Results

### Activity of Anti-CTLA-4 and anti-PD-1 and Their Combination in Syngeneic Mouse Tumor Models

We began preclinical assessment of anti-CTLA-4 and anti-PD-1 combination therapy in mouse syngeneic tumor models. While anti-PD-1 functions solely as an antagonist, we and others have shown that anti-CTLA-4 efficacy in preclinical and clinical settings is at least partially dependent on reduction of intratumoral Tregs [13–15]. We treated mice with a non-depleting mIgG1 isotype anti-PD-1 antibody (4H2) and with mIgG2b isotype anti-CTLA-4 (9D9), which most closely resembles the hIgG1 isotype of ipilimumab in its capacity to elicit antibody-dependent cell-mediated cytotoxicity (ADCC) [23].

**Fig 1:**
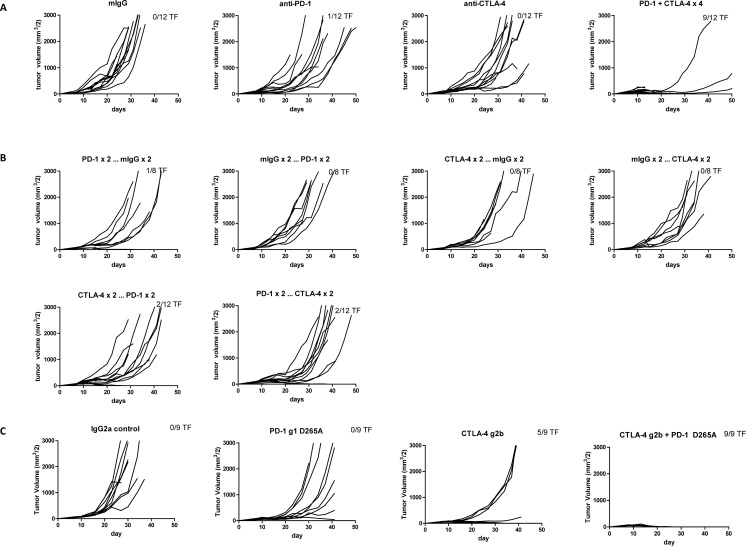
Antitumor Responses of Anti-CTLA-4 and Anti-PD-1 Antibodies in Staged MC38 and CT26 Tumor Models. A-B. Groups of 8–12 C57/BL6 mice were sourced from Taconic and subcutaneously injected with 2×10^6^ MC38 cells. After tumors were measured on day 7, mice were randomized (58 mm^3^ mean tumor volume per group) and then treated with the designated mAb (200 μg/dose IP) followed by additional doses on days 10, 14, and 17. A. Groups were treated with 4 doses of single or combined agents. Anti-PD-1 vs control p = 0.0176; anti-PD-1 and anti-CTLA-4 vs control p< 0.0001. B. Sequential dosing, where 4 doses were given as 2 doses of one mAb followed by 2 doses of the other mAb and the converse. Anti-CTLA-4 followed by anti-PD-1 vs control p = 0.0250; anti-PD-1 followed by anti-CTLA-4 vs control p = 0.0015. Tumor volumes were measured twice weekly. The number of tumor-free (TF) mice per group is indicated. C-D. Groups of 10 BALB/c mice sourced from CRL (C) or HAR (D) Laboratories were subcutaneously injected with 1×10^6^ CT26 cells. After tumors were measured on day 7, mice were randomized (C: 56 mm^3^ and D: 35 mm^3^ mean tumor volume) and then treated with the designated mAb (200 μg/dose IP) followed by additional doses on days 10, 14 (HAR mice), or 10, 14, 17 (CRL mice). Anti-CTLA-4 vs control p = 0.0035; anti-CTLA-4 and anti-PD-1 vs control p<0.0001. Tumor volumes were measured twice weekly. The number of TF mice per group is indicated.

In a therapeutic treatment model of MC38 colon adenocarcinoma, monotherapy with either anti-CTLA-4 or anti-PD-1 was partially efficacious, with anti-PD-1 eliciting greater rates of tumor rejection (Fig 1A). Consistent with earlier reports [24, 25], combination treatment promoted even greater antitumor activity, with rapid tumor rejection and durable antitumor immunity observed in a majority of mice (Fig 1A).

In the CT26 colon carcinoma model, anti-PD-1 and anti-CTLA-4 combination therapy also elicited tumor rejections that were superior to the individual therapies (Fig 1C and 1D). Notably, antitumor efficacy varied by the source of the mice as tumor-bearing mice from HAR were more responsive to therapy than those from CRL, possibly as a result of intestinal microbiome differences [26, 27] (Fig 1C and 1D). Interrogation of other tumor models such as RENCA renal carcinoma and B16F10 melanoma demonstrated no single agent activity and little or no combinatorial activity.

In general, the response of different types of tumors to checkpoint blockade is more closely associated with inherent immunogenicity (mutational burden or dominant neoantigens) than with the tissue of tumor origin [28]. For example, CTLA-4 and PD-1 blockade are ineffective in the B16 mouse melanoma tumor model yet have demonstrated activity in human melanoma. Similarly, the antitumor activity observed with anti-PD-1 mAb treatment in mouse colon carcinoma models shown here is not observed in human colorectal cancer patients except for patients with microsatellite unstable tumors [29] or mismatch repair deficient tumors [30].

Alternative dosing schedules in the MC38 model were evaluated to determine their impact on the efficacy of combination immunotherapy. Interestingly, when mAb treatments were staggered (two doses of anti-PD-1 followed sequentially by two doses of anti-CTLA-4 or *vice versa*), antitumor activity was largely muted (Fig 1B). Pharmacokinetics did not account for the loss of activity since both antibodies have similar half-lives of ~7 days [13]. A “reciprocal” dosing schedule was also evaluated in which the dose of one antibody was fixed while the other was titrated. When anti-PD-1 mAb was dosed at 10 mg/kg Q3D×3, addition of anti-CTLA-4 at the lower doses of 20 or 60 μg (1 or 3 mg/kg, respectively) resulted in reduced antitumor activity (Table 1). In contrast, when CTLA-4 mAb was dosed at 10 mg/kg Q3D×3, antitumor activity was sustained at lower doses (1 or 3 mg/kg) of anti-PD-1 mAb. A requirement for simultaneous co-blockade of both CTLA-4 and PD-1 in effector T cells (Teffs) may be one explanation for the superiority of concurrent over sequential treatment. Indeed, CTLA-4 and PD-1 co-expression was detected in approximately 72% of AH1 antigen-specific CD8^+^ T cells in CT26 tumors (S2A Fig). In addition, approximately 24% of polyclonal tumor-infiltrating CD8^+^ T cells in MC38 tumors were double positive (S2B Fig) supporting the notion that these two receptors could directly collaborate in the suppression of Teff responses.

**Table 1:** Reciprocal Dosing of Anti-PD-1 and Anti-CTLA-4 Antibodies in a Staged MC38 Tumor Model.

Group	Anti-PD-1 (μg)	Anti-CTLA-4 (μg)	% TF Mice	% TGI at Day 22
1	0	0	0	0
2	200	0	0	51
3	200	20	7	68
4	200	60	13	82
5	200	200	47	91
6	60	200	60	91
7	20	200	53	94
8	0	200	0	55

15 mice/group C57BL/6 mice were subcutaneously injected with 2×10^6^ MC38 cells. On day 8 tumors were measured and mice randomized to treatment groups (average tumor volume was ~70 mm^3^). On days 8, 11, and 15, mice in Groups 2–8 were treated with the designated mAbs and Group 1 was injected with 400 μg of isotype control (anti-DT mAb mIgG1; BMS); all groups had a total of 400 μg of total antibody injected, with the balance provided by the isotype control. Tumor volumes were measured twice weekly. The percentage of TF mice per group and the percentage tumor growth inhibition (TGI) are indicated. All combination groups (3–7) exhibited increased TGI relative to the control group (1, p<0.01), whereas both single agent groups (2 and 8) did not.

Next we confirmed the role of CD4^+^ and CD8^+^ T cells in anti-PD-1 and anti-CTLA-4 treatment of MC38 tumor-bearing mice (S3 Fig). As expected, co-administration of CD8-depleting antibodies with the initial doses of anti-CTLA-4 and anti-PD-1 abrogated antitumor responses in all treatment groups. In contrast, depletion of CD4^+^ T cells enhanced antitumor responses in tumor-bearing mice treated with control and therapeutic mAbs, likely due to the loss of intratumoral Tregs [31].

### Pharmacodynamic Effects of Combination Therapy on the Mouse Tumor Microenvironment and Infiltrating Lymphocytes

**Fig 2:**
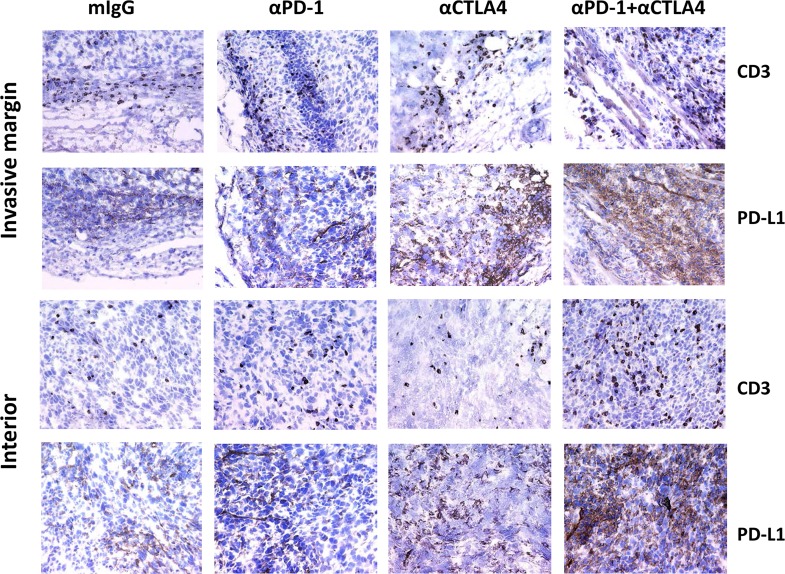
Immunohistochemistry of MC38 Tumors: Detection of CD3^+^ T Cells and PD-L1^+^ Cells on the Tumor Perimeter and Interior. C57BL/6 mice bearing MC38 tumors (n = 4–5 mice/group) were treated on days 7, 10, and 13 with the indicated antibodies. Tumors were harvested on day 14 and processed for IHC. Sections were stained with anti-CD3 (Abcam SP7 ab16669) or anti-PD-L1 mAbs (14–5982). IHC images of tissue sections derived from the periphery of the tumor (invasive margin) or from within the tumor (interior) are shown.

PD-L1 expression and CD8^+^ T cell tumor infiltration are key markers of tumor sensitivity and responsiveness to immunotherapies [32–34]. Immunohistochemistry (IHC) was employed to examine MC38 tumors for PD-L1 expression and CD3^+^ T-cell infiltration after treatment. In control mIgG1-treated mice, CD3^+^ T cells were largely restricted to the periphery of the tumor at the invasive margin (Fig 2) and were coincident with PD-L1 expression. Treatment with anti-PD-1 and anti-CTLA-4 as single agents resulted in T-cell infiltration into tumors; when anti-PD-1 and anti-CTLA-4 were combined, even greater numbers of CD3^+^ T cells and PD-L1^+^ cells were found within the tumor mass (Fig 2). In tumors from mice treated with the combined agents, the ratio of intratumoral to peripheral tumor CD3^+^ T cells increased 475% (p = 0.03 as compared to the isotype control) based on mean cell counts from three interior fields (range: 65–569 CD3^+^ T cells in combined group, 18–99 CD3^+^ T cells in control group) and three invasive margin fields (range: 26–208 CD3^+^ T cells in combined group, 24–192 CD3^+^ T cells in control group) per mouse (n = 5 mice/group). IFN-γ-deficient mice failed to respond similarly (S4 Fig), and we found elevated levels of IFN-γ, IL-10, and IL-13 (among 15 examined) present in tumor lysate supernatants from wild-type mice treated with both antibodies than with either antibody alone (S5 Fig). *In vitro*, IFN-γ stimulation induced upregulation on PD-L1 and MHC-I but not PD-L2 on MC38 tumor cells (S6 Fig). These findings support the notion that treatment with both anti-PD-1 and anti-CTLA-4 antibodies elicits more robust antitumor immune responses, largely driven by IFN-γ and T cells, than can be elicited by either antibody alone.

**Fig 3:**
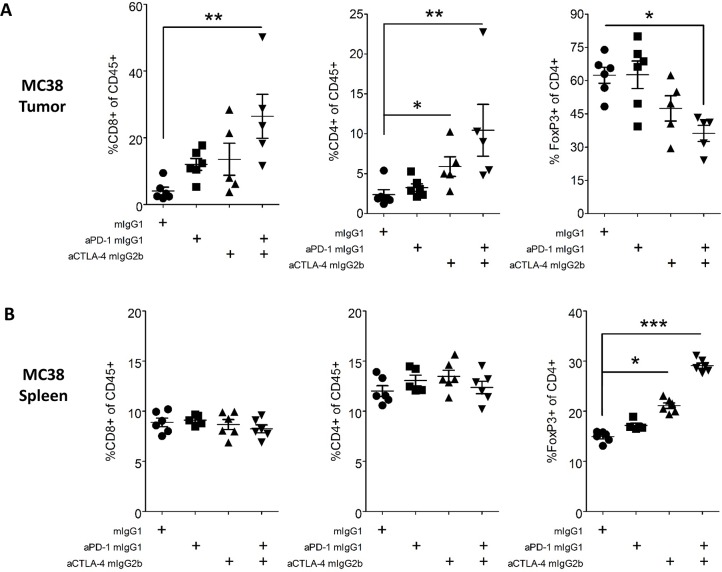
FACS Analysis of Tumor and Spleen T Cell Populations from MC38 Tumor-Bearing Mice. MC38 colon tumor cells (2×10^6^) were implanted subcutaneously into female C57BL/6 mice. At day 7 post implantation, tumor-bearing mice were randomized and dosed with 10 mg/kg of antibody IP Q3D×3. On day 15 post implantation, tumors were harvested, dissociated into single-cell suspensions, and stained for flow cytometry. Data are representative of three MC38 independent experiments with ≥5 mice/group/experiment. A-B. Percentages of CD45^+^ cells that were CD8^+^ Teffs, CD4^+^, FoxP3- Teffs, and CD4^+^ FoxP3^+^ Tregs found in tumor (A) and matched spleen (B).

Flow cytometry was used to directly assess tumor-infiltrating leukocytes (TIL) in these models. In MC38 tumor-bearing mice, treatment with either anti-CTLA-4 or anti-PD-1 antibodies only modestly increased the frequency of effector CD8^+^ and CD4^+^ T cells amongst TIL, whereas combination treatment had a more significant effect (550% and 330% increases, respectively, relative to control treatment, p<0.01, Fig 3A). There was a concurrent decrease in the frequency of Tregs amongst CD4^+^ TIL in mice receiving combination treatment (45% decrease, p<0.01, Fig 3A). In contrast to tumors, analysis of splenic T cells showed no changes in CD8^+^ or non-regulatory CD4^+^ T cells, although treatment with either antibody alone or in combination led to an increased frequency of Tregs (Fig 3B), as has previously been reported following anti-CTLA-4 treatment [35].

**Fig 4:**
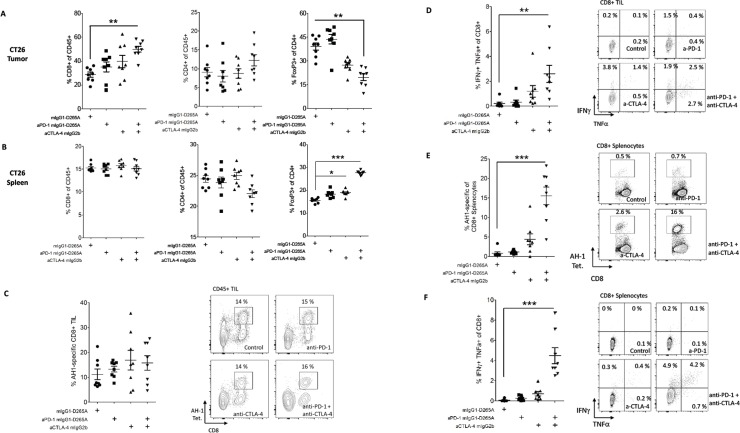
FACS Analysis of Tumor and Spleen T Cell Populations from CT26 Tumor-Bearing Mice. CT26 colon tumor cells (1x10^6^) were implanted subcutaneously into female BALB/c mice. At day 7 post implantation, tumor-bearing mice were randomized and dosed with 10 mg/kg of antibody IP Q3D×3. On day 15 post implantation, tumors were harvested, dissociated into single-cell suspensions, and stained for flow cytometry (n = 8/group); data are representative of two independent experiments. Cytokine production data are representative of one experiment utilizing *ex vivo* ICS and one experiment utilizing ICS following peptide re-stimulation. A-B. Percentages of polyclonal CD8^+^ T cells of CD45^+^ cells (left), CD4^+^ T cells of CD45^+^ cells, or FoxP3^+^ cells of CD4^+^ T cells (right) in tumors (A) and spleens (B) after the indicated treatments. C. Representative frequencies and plots and of AH1-specific (tetramer^+^) CD8^+^ T cells as a percentage of total TIL. D. Representative frequencies and plots of IFN-γ/TNF-α^+^ cells of total tumor-infiltrating CD8^+^ T cells following *in vitro* AH1 peptide stimulation. E. Representative frequencies and plots of AH1-specific (tetramer^+^) CD8^+^ T cells as a percentage of total splenic CD8^+^ T cells. F. Representative frequencies and plots of IFN-γ/TNF-α^+^ cells of total splenic CD8^+^ T cells following *in vitro* AH1 peptide stimulation. Error bars depict the SEM.

In the CT26 model, tumor-bearing mice treated with anti-CTLA-4 in combination with anti-PD-1 exhibited an increase in the frequency of CD8^+^ T cells, but not of CD4^+^ T cells amongst TIL (72% increase in CD8^+^ T cell frequency relative to control treatment, p<0.01, Fig 4A). Similarly, the frequency of tumor-infiltrating Tregs was decreased in mice receiving combination treatment (50% reduction, p<0.01, Fig 4B). Neither pharmacodynamic effect was seen in mice treated with anti-PD-1 alone, consistent with the lack of activity of anti-PD-1 monotherapy in CT26 (Fig 4C), or in mice treated with anti-CTLA-4 alone. In the spleens of CT26 tumor-bearing mice, treatment had no significant effect on total CD8^+^ or CD4^+^ T cell frequencies, although anti-CTLA-4 alone or in combination with anti-PD-1 did lead to increased frequencies of Tregs as a percentage of total CD4^+^ T cells (p<0.05, Fig 4D). To track tumor antigen-specific T cell responses in this model, we utilized AH1 peptide MHC class I tetramers and peptide re-stimulation. In tumors, tetramer^+^ (AH1-specific) CD8^+^ T cells constituted approximately 15% of infiltrating leukocytes; this frequency was not consistently altered by treatment with either anti-PD-1 or anti-CTLA-4 (Fig 4E). However, *in vitro* peptide stimulation of TIL did reveal a potent synergistic enhancement of CD8^+^ TIL effector function by anti-PD-1 and anti-CTLA-4 antibodies (>10-fold increase in the frequency of IFN-γ^+^ TNF-α^+^ CD8^+^ T cells relative to control, p<0.001, Fig 4F and S7 Fig), consistent with previous reports [25].

AH1-specific CD8^+^ T cells were also expanded in the spleens of CT26 tumor-bearing mice treated with anti-PD-1 and anti-CTLA-4, increasing from approximately 1% to an average of 15.6% of splenic CD8^+^ T cells (p<0.001, Fig 4E). An increase of similar magnitude was seen in the frequency of splenic CD8^+^ T cells producing IFN-γ and TNF-α in response to AH1 peptide stimulation (p<0.001, Fig 4F and S7 Fig).

**Fig 5:**
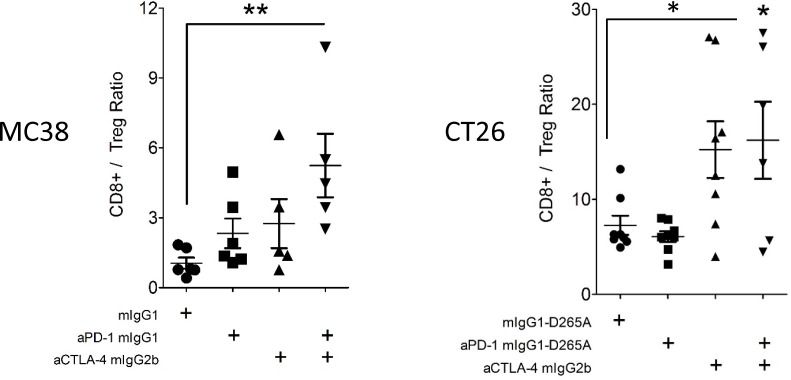
CD8^+^:Treg TIL Ratios in Treated MC38 and CT26 Tumor-Bearing Mice. Mice were implanted with MC38/CT26 tumor cells, treated, and analyzed as in Fig 3 and Fig 4. Ratios of CD8^+^ Teff / CD4^+^ Treg tumor-infiltrating T cells found in MC38 (left) and CT26 (right) tumors. Error bars depict the SEM.

The ratio of tumor-infiltrating CD8^+^ T cells to tumor-infiltrating Tregs was used to assess the state of antitumor T cell responses. In the MC38 model, anti-PD-1 and anti-CTLA-4 combination therapy, but neither monotherapy significantly augmented this ratio (p<0.01, Fig 5). In the CT26 model, which is partially responsive to anti-CTLA-4 monotherapy, the CD8^+^/Treg TIL ratio was significantly increased in mice receiving the combination therapy and trended higher in mice receiving anti-CTLA-4 alone (p<0.05, Fig 5). Taken together, these data demonstrated clear enhancement of mouse antitumor T cell responses with anti-PD-1 and anti-CTLA-4 combination treatment.

### *In Vitro* Activity of Ipilimumab and Nivolumab in Human T Cells

**Fig 6:**
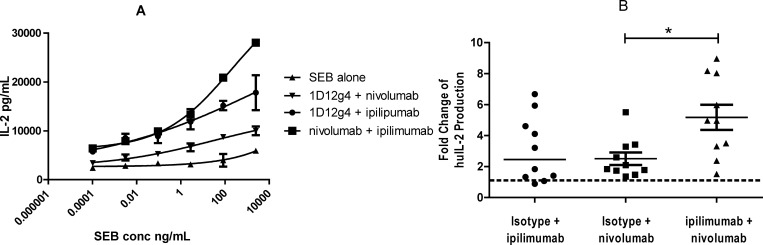
Ipilimumab (Anti-CTLA-4) and Nivolumab (Anti-PD-1) Antibodies Potentiate IL-2 Secretion in SEB-Stimulated Human PBMC. Frozen PBMC from 10 normal healthy donors were seeded at 1×10^5^ cells/well and stimulated with SEB titrated 30-fold from 2.5 μg/mL. Ipilimumab, nivolumab, or isotype control antibody (1D12g4) were present at 10 μg/mL each. IL-2 secretion was measured by ELISA (in triplicate) on day 3. A. Dose titration data from one donor. B. Pooled data from 10 donors are plotted as fold-change over IL-2 values for SEB at 85 ng/mL. Bar = two-tailed T test p = 0.0088.

The combination activity of the clinical anti-human CTLA-4 and PD-1 antibodies, ipilimumab and nivolumab [36, 37], were assessed in *in vitro* functional assays. In SEB-stimulated PBMC cultures, the addition of either nivolumab or ipilimumab alone enhanced IL-2 production over baseline levels, while the combination of both antibodies stimulated still higher IL-2 release (Fig 6). At a representative SEB concentration of 2.5 μg/mL, ipilimumab and nivolumab each promoted a mean 2-fold increase in IL-2 production, while their combination was 5-fold more potent than baseline (Fig 6B).

**Fig 7:**
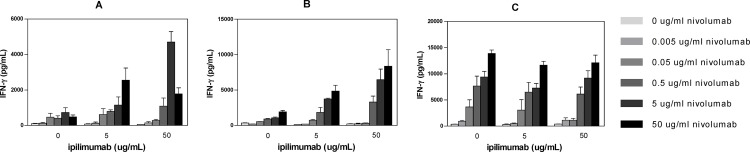
Ipilimumab (Anti-CTLA-4) and Nivolumab (Anti-PD-1) Antibodies Potentiate IL-2 Release in an Allogeneic MLR Assay. Allogeneic mixed lymphocyte response (MLR) assays were performed using monocyte-derived DC and CD4^+^ T cells from 3 donors at a ratio of 1:10 (DC:T cells). CD4^+^ T cells and DC were incubated for 6 days in the presence of titrated nivolumab with 0, 5, or 50 μg/mL ipilimumab. Culture supernatants were harvested on day 5 for enzyme-linked immunosorbent assay (ELISA) analysis of IFN-γ secretion.

In an allogeneic T:DC MLR assay, ipilimumab treatment alone did not augment the T-cell response as measured by IFN-γ production or proliferation (Fig 5). Nivolumab was highly active in this assay, as previously described [37]. While T-cell activity was observed with nivolumab as a single agent, its combination with ipilimumab further enhanced IFN-γ production in two of three donors tested when the concentration of nivolumab was ≥0.5 μg/mL (Fig 7).

Lastly, we performed cytokine release assays to evaluate ipilimumab- and nivolumab-mediated cytokine secretion by naive human peripheral blood cells in the absence of T cell receptor (TCR) stimulation. While an anti-CD3 agonist antibody (UCHT-1) efficiently induced cytokine secretion by these cells, the addition of ipilimumab and/or nivolumab to healthy donor whole blood at concentrations of up to 100 μg/mL did not induce any meaningful cytokine secretion. These data suggested that ipilimumab and nivolumab treatments could collaboratively enhance effector and memory T cell responses without inappropriately activating naive T cells.

### Cynomolgus Macaque Toxicology

Ipilimumab and nivolumab therapy in humans can result in the induction of inflammatory AEs affecting multiple organ systems [21, 38, 39]. To assess the potential safety of combination therapy in the clinic, we conducted a cynomolgus macaque toxicology study. Three groups of animals (5 male and 5 female monkeys per group) consisting of a control group, a low-dose combination group (3 mg/kg ipilimumab and 10 mg/kg nivolumab), and a high-dose combination group (10 mg/kg ipilimumab and 50 mg/kg nivolumab) were treated once weekly for 4 weeks. Six animals per group were necropsied on day 30, while the remainder of the animals (recovery group) was analyzed at day 59.

CD4 and CD8 lymphocyte subsets, B lymphocytes, NK cells, and monocytes in cynomolgus PBMC were analyzed by flow cytometry on days 7, 28, and 58 and no significant alterations of lymphocyte subpopulations were observed. Highly variable increases in T helper lymphocytes were detected in the high-dose animals as compared to the untreated control group that reverted to baseline averages at day 58.

In the high-dose combination animals, 40% showed recurrent diarrhea as compared to 20% of animals in the low-dose group. Decreased food consumption was also observed in 20% of the high-dose group. A single male monkey in the high-dose group was found dead on day 23. The animal’s early death was attributed to acute gastric dilatation (bloat) based on gross and microscopic findings by the CRL veterinary pathologist. The underlying cause of bloat could not be determined, and therefore a possible direct or indirect relationship to test articles could not be ruled out.

Combination treatment-related effects were observed in the spleen (increased weight and lymphoid follicle hypertrophy and/or marginal zone expansion), and lymph nodes (decreased germinal centers and/or hypocellularity), and were dose dependent. In most high-dose and some low-dose monkeys, gastrointestinal tract inflammation was detected in the lamina propria and was associated with decreased albumin, increased globulin, and/or increased neutrophil and eosinophil counts, and mononuclear inflammation of the large intestine. Acinar cell degranulation was seen in the pancreas of the high-dose group and was attributable to reduced food intake. Toxicology results are summarized in Table 2. Given that similar inflammatory events were not observed in cynomolgus monkeys treated with ipilimumab [36] or nivolumab [37] alone, these findings suggested that combination therapy may further exacerbate self-reactive immune responses in patients.

**Table 2:** Cynomolgus Toxicology Signals with Ipilimumab and Nivolumab Combination.

Group	M/F	Treatment	Dose	Diarrhea^a^	Mean Spleen Weight^b^ (g)		Spleen Pathology^c^	Gastrointestinal Pathology^d^
			mg/kg	n/N	Day 30 M/F	Day 59 M/F	n/N	n/N
1	5/5	saline control	—	0/10	3.9/2.8	3.5/3.7	0/6	0/6
2	5/5	nivolumab + ipilimumab	10 3	2/10	4.0/3.6	4.3/2.4	2/6	2/6
3	5/5	nivolumab + ipilimumab	50 10	4/10	6.1/4.47	7.5/3.2	4/5	3/5

^a^ Incidence of repeated diarrhea (number of animals with finding/number of animal examined).

^b^ Mean spleen weight on days 30 and 59; at day 30, 3 monkeys per sex per group with the exception of 2 males in Group 3; at day 59, 2 monkeys per sex per group.

^c^ Incidence of lymphoid follicle hypertrophy or marginal zone expansion: number of animals with finding (n) / number of animals examined (N).

^d^ Minimal, diffuse lymphoplasmacytic inflammation in the lamina propria with concurrent enlargement of the colonic or pelvic lymph nodes: number of animals with finding (n) / number of animal examined (N).

## Discussion

Checkpoint inhibitor antagonists have transformed the therapeutic landscape for many cancers, and a growing body of evidence indicates that combination therapies have the potential to confer even greater benefits [5]. The two most advanced tumor immunotherapy antagonists target CTLA-4 and PD-1, which promote negative regulation through distinct but complementary signaling pathways in Teffs [40–42]. Co-expression of CTLA-4 and PD-1 has been detected in HCV-specific T cells [43] as well as in melanoma TIL [44]. In the most comprehensive clinical trial to date, Larkin et al [21] explored ipilimumab and nivolumab and their combination in a double-blinded phase 3 study in previously untreated patients with advanced melanoma. The combination showed an objective response rate of 57.6% compared to 43.7% and 19% with nivolumab and ipilimumab monotherapies, respectively, along with a superior progression-free survival of 11.5 months for the combination. These findings in metastatic melanoma have led to the testing of the combination in multiple malignancies in a variety of dosing schedules [39, 45–48].

Here we present the preclinical rationale that led to the combination of ipilimumab and nivolumab immunotherapies. Antibodies to CTLA-4 and anti-PD-1 showed enhanced antitumor activities when used in combination in two mouse syngeneic tumor models. Immunohistochemistry, flow cytometry, and cytokine analysis were used to interrogate the changes in the tumor microenvironment in response to antibody therapy. In the IHC analysis of day 15 MC38 tumors from control IgG-treated mice, CD3^+^ T cells largely localized around tumors. These IHC images were remarkably similar to those described for human tumors where T cells accumulated at the invasive margins [49–51]. PD-L1 expression was observed to be coincident with T cells surrounding the tumors and likely resulted from local IFN-γ expression after T cell recognition of tumor antigen [50]. Consistent with this suggestion, IHC analysis of MC38 tumors in IFN-γ-deficient mice showed little PD-L1 expression. More T cells were detected within the tumor mass after combination treatment, suggesting that either T cells acquired the ability to migrate into the tumor mass and/or expanded within the tumor.

The potency of combined anti-CTLA-4 and anti-PD-1 is likely due to the effect of the antibodies on different cell populations within the tumor. Treg depletion at the tumor site is a requisite part of the antitumor activity of anti-CTLA-4 whose potency varies by antibody Fc isotype [13]. CTLA-4 blockade in concert with Treg depletion enhances the function and number of CD4^+^ and CD8^+^ T cells. Anti-PD-1, which blocks PD-1 interactions with its ligands, functions in the absence of FcαR binding [52]. Anti-PD-1 monotherapy results in modest increases in CD8^+^ T cells in responsive tumors (herein, [53]). Using anti-CTLA-4 as a mIgG2b isotype, a partial reduction of Tregs at the tumor site was observed in MC38 and CT26 tumors in mice treated with both anti-CTLA-4 and anti-PD-1, and was accompanied by the expansion of tumor-infiltrating CD8^+^ T cells. An enhanced CD8^+^ Teff to Treg ratio is considered a favorable correlate of antitumor activity [35] and the combination therapy in these preclinical studies resulted in a higher ratio than the single agents. Interestingly, combination therapy also resulted in a large expansion of cytokine-competent AH1-specific CD8^+^ T cells in the spleens of CT26 tumor-bearing mice. This novel finding suggests that anti-CTLA-4 and anti-PD-1 act to enhance the priming of new antitumor responses and/or the persistence of existing responses.

Cytokine analysis at the MC38 tumor site revealed that IFN-γ was secreted at higher levels after CTLA-4 or PD-1 treatment compared to isotype control mAb, and IFN-γ secretion was further enhanced when the mAbs were combined. Similarly, in the CT26 tumors, the competency of AH1 tumor antigen-specific CD8^+^ T cells to produce IFN-γ and TNF-α was increased with combination treatment relative to control or single agent-treated mice. The importance of IFN-γ in antitumor activity was also confirmed by absence of activity in IFN-γ-receptor knock-out (KO) mice (herein, [54]). Levels of IL-13 were also increased with PD-1 and combination treatment. In support of a role for IL-13 in promoting antitumor activity, P815 cells expressing IL-13 grew slower than parental tumor cells [55]. IL-10, which is normally associated with reduced antitumor activity [56] was also increased. Of note, Mumm et al [57] reported a positive role for IL-10 in expanding tumor CD8^+^ cells.

Ipilimumab and nivolumab therapy in humans is characterized by the induction of inflammatory AEs that affect multiple organ systems [21, 38, 39]. Although there are no new types of AEs detected in patients treated with the combination, there is an increased frequency of AEs leading to discontinuation of therapy. These can be controlled by steroids and other agents that do not impact antitumor activity. Notably, similar inflammatory events were not observed in cynomolgus monkeys treated with ipilimumab [36] or nivolumab [37] as monotherapies. However, the combined use of ipilimumab and nivolumab in a cynomolgus macaque toxicity study resulted in a dose-dependent increase in AEs characterized by diarrhea and weight loss. These symptoms were demonstrative of gastrointestinal inflammation, as subsequently confirmed by pathological findings, and suggest that combination therapy may lead to enhanced inflammation or immune activation as a result of self-reactivity. In mice, we found that antitumor potency could be maintained in mice treated with fixed dose anti-CTLA-4 and titrated doses of anti-PD-1 or, to a lesser extent, with fixed-dose anti-PD-1 and titrated doses of anti-CTLA-4. These observations support further exploration of ipilimumab and nivolumab dosing regimens that will maintain efficacy while minimizing AEs and patient discontinuation in clinical trials.

It is noteworthy that a significant percentage of tumor tetramer^+^ CD8^+^ T cells were double positive, suggesting that the simultaneous blockade of the two receptors results in functionality that is superior to singular blockade. Combination of CTLA-4 and PD-1 blockade has only been found effective in a subset of syngeneic tumor models; non-responsive models may require additional therapies such as vaccines or radiation to elicit tumor-reactive T cells. Similarly, human cancers that are not responsive to anti-PD-1 and/or anti-CTLA-4 may require additional immune modulation if patients are to be converted to durable responders [5].

## Supporting Information

S1 Fig Binding and Blocking Characteristics of Anti-PD-1 Antibody. A. Binding of anti-PD-1 mAb to murine PD-1 transfectants. Anti-PD-1 mAb was serially diluted and incubated with CHO-PD-1 cells followed by detection with a FITC-conjugated secondary to mouse IgG Fcγ. B. Inhibition of PD-L1-Fc binding to PD-1 CHO transfectants. CHO-PD-1 cells were preincubated with titrated anti-PD-1 mAb followed by addition of PD-L1-Fc at 2 μg/mL. Cell-bound PD-L1-Fc was detected with a FITC-conjugated secondary to human IgG Fcγ. C. Inhibition of PD-L2-Fc binding to PD-1 CHO transfectants CHO-PD-1 cells were preincubated with titrated anti-PD-1 antibody followed by addition of PD-L2-Fc at 15 μg/mL. Cell-bound PD-L-Fc was detected with a FITC-conjugated secondary to human IgG Fcγ.
(TIFF)

S2 Fig PD-1/CTLA-4 Co-Expression by Tumor-Infiltrating CD8^+^ T Cells in the CT26 and MC38 Models. A. TIL from CT26 tumor-bearing mice were harvested 15 days after tumor implantation. On left, frequencies of PD-1/CTLA-4 co-expression in each population are shown. *** p<0.001. On right, representative overlaid plot of PD-1/CTLA-4 (ICS) co-expression in AH-1 tetramer^+^ CD8^+^ TIL (red) and AH-1 tetramer- CD8^+^ TIL (polyclonal, blue). B. PD-1 and CTLA-4 co-expression on polyclonal CD8+ T cells harvested from MC38 tumors 15 days after implantation.
(TIFF)

S3 Fig CD4 Depletion Increases and CD8 Depletion Decreases Antitumor Activity of Treatment Antibodies. A. C57BL/6 mice were with injected 2×10^6^ MC38 cells and treated on days 7, 10, and 13 with 400 μg of isotype control or single agent, or 200 μg of each agent for the combination therapy. Anti-PD-1 vs control p = 0.0013; anti-PD-1 and anti-CTLA-4 vs control p<0.0001. B. Treatment of mice as in A with the addition of 500 μg of depleting CD4 mAb (GK1.5 BioXCell) i.p. on day 7. Anti-PD-1 vs control p = 0.0001; anti-PD-1 and anti-CTLA-4 vs control p<0.0001. C. Treatment of mice as in A with the addition of 500 μg of depleting CD8 mAb (53.6.72 BioXCell) i.p. on day 7. Anti-PD-1 and anti-CTLA-4 vs control p = 0.0149. The number of tumor-free (TF) mice per group is shown. FACS analysis of group B and C blood samples confirmed >90% depletion of CD4^+^ and CD8^+^ T cells four days after administration of depleting antibodies.
(TIFF)

S4 Fig Antitumor Activities of Therapeutic Antibodies Depend on Interferon-Gamma. Wild-type (A) or B6.129S7-*Ifng*^*tm1Ts*^/J (B) C57BL/6 mice were injected with 2×10^6^ MC38 tumor cells and treated with 400 μg of isotype IgG1 or 200 μg of isotype with 200 μg of anti-PD-1 or anti-CTLA-4, or 200 μg each of anti-PD-1 and anti-CTLA-4 for the combination therapy on days 0, 4, and 7. The number of tumor-free (TF) mice per group is shown. A. anti-PD-1 vs control p = 0.0082; anti-CTLA-4 vs control p = 0.0464; anti-PD-1 and anti-CTLA-4 vs control p<0.0001. B. anti-PD-1 and anti-CTLA-4 vs control p = 0.0264.
(TIFF)

S5 Fig Enhancement of Intratumoral Cytokine Secretion by Therapeutic Antibodies. C57BL/6 mice were injected with 2×10^6^ MC38 cells and treated on days 7, 10, and 13 with 200 μg of isotype control or single agents, or 200 μg of each agent for the combination therapy. On day 15 post implantation, tumors were harvested, manually dissociated into single-cell suspensions, and levels of intratumoral cytokines were assessed via bead-based cytokine arrays (FlowCytomix, eBioscience). Results are shown for IFN-γ, IL-10, and IL-13.
(TIFF)

S6 Fig Interferon Gamma Upregulates PD-L1 and MHC I, But Not PD-L2 or MHC II on MC38 Tumor Cells. MC38 cells were cultured in the presence of recombinant murine IFN-γ (R&D Systems) at 5 ng/mL for 24 hours and analyzed by FACS for expression of mouse PD-L1 (eBioscience, MIH5), mouse PD-L2 (eBioscience, 122), mouse MHC I (H-2D^b^; eBioscience 28-14-8), or mouse MHC II (I-A/I-E; eBioscience M5/114.15.2).
(TIFF)

S7 Fig Cytokine Production by CT26 TIL and Splenic CD8^+^ T Cells Following Peptide Stimulation *In Vitro*. CT26 colon tumor cells (1x10^6^) were implanted subcutaneously into BALB/c mice, which were then treated and analyzed as described in Fig 3. A. Frequencies of IFN-γ^+^ TNF-α^-^ and IFN-γ^-^ TNF-α^+^ cells of total tumor-infiltrating CD8^+^ T cells following *in vitro* AH1 peptide stimulation. B. Frequencies of IFN-γ^+^ TNF-α^-^ and IFN-γ^-^ TNF-α^+^ cells of total splenic CD8^+^ T cells following *in vitro* AH1 peptide stimulation. * p<0.05; ** p<0.01; *** p<0.001.
(TIFF)
