# Correction: Preclinical Development of Ipilimumab and Nivolumab Combination Immunotherapy: Mouse Tumor Models, In Vitro Functional Studies, and Cynomolgus Macaque Toxicology

**DOI:** 10.1371/journal.pone.0167251

**Published:** 2016-11-18

**Authors:** Mark J. Selby, John J. Engelhardt, Robert J. Johnston, Li-Sheng Lu, Minhua Han, Kent Thudium, Dapeng Yao, Michael Quigley, Jose Valle, Changyu Wang, Bing Chen, Pina M. Cardarelli, Diann Blanset, Alan J. Korman

Fig 1D is missing from Fig 1. Please see the corrected Fig 1 here.

**Fig 1:**
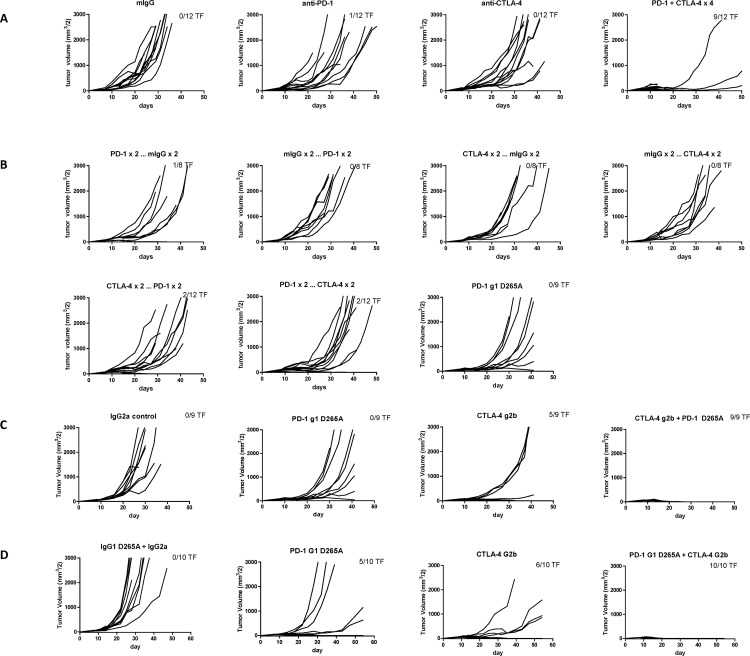
Antitumor Responses of Anti-CTLA-4 and Anti-PD-1 Antibodies in Staged MC38 and CT26 Tumor Models. A-B. Groups of 8–12 C57/BL6 mice were sourced from Taconic and subcutaneously injected with 2×10^6^ MC38 cells. After tumors were measured on day 7, mice were randomized (58 mm^3^ mean tumor volume per group) and then treated with the designated mAb (200 μg/dose IP) followed by additional doses on days 10, 14, and 17. A. Groups were treated with 4 doses of single or combined agents. Anti-PD-1 vs control p = 0.0176; anti-PD-1 and anti-CTLA-4 vs control p< 0.0001. B. Sequential dosing, where 4 doses were given as 2 doses of one mAb followed by 2 doses of the other mAb and the converse. Anti-CTLA-4 followed by anti-PD-1 vs control p = 0.0250; anti-PD-1 followed by anti-CTLA-4 vs control p = 0.0015. Tumor volumes were measured twice weekly. The number of tumor-free (TF) mice per group is indicated. C-D. Groups of 10 BALB/c mice sourced from CRL (C) or HAR (D) Laboratories were subcutaneously injected with 1×10^6^ CT26 cells. After tumors were measured on day 7, mice were randomized (C: 56 mm^3^ and D: 35 mm^3^ mean tumor volume) and then treated with the designated mAb (200 μg/dose IP) followed by additional doses on days 10, 14 (HAR mice), or 10, 14, 17 (CRL mice). Anti-CTLA-4 vs control p = 0.0035; anti-CTLA-4 and anti-PD-1 vs control p<0.0001. Tumor volumes were measured twice weekly. The number of TF mice per group is indicated.
