# Supplementary figures and images for: Preclinical Development of Ipilimumab and Nivolumab Combination Immunotherapy: Mouse Tumor Models, In Vitro Functional Studies, and Cynomolgus Macaque Toxicology
Source: PLoS One. 2016 Sep 9;11(9):e0161779. doi: 10.1371/journal.pone.0161779 (PMC5017747; doi:10.1371/journal.pone.0161779)

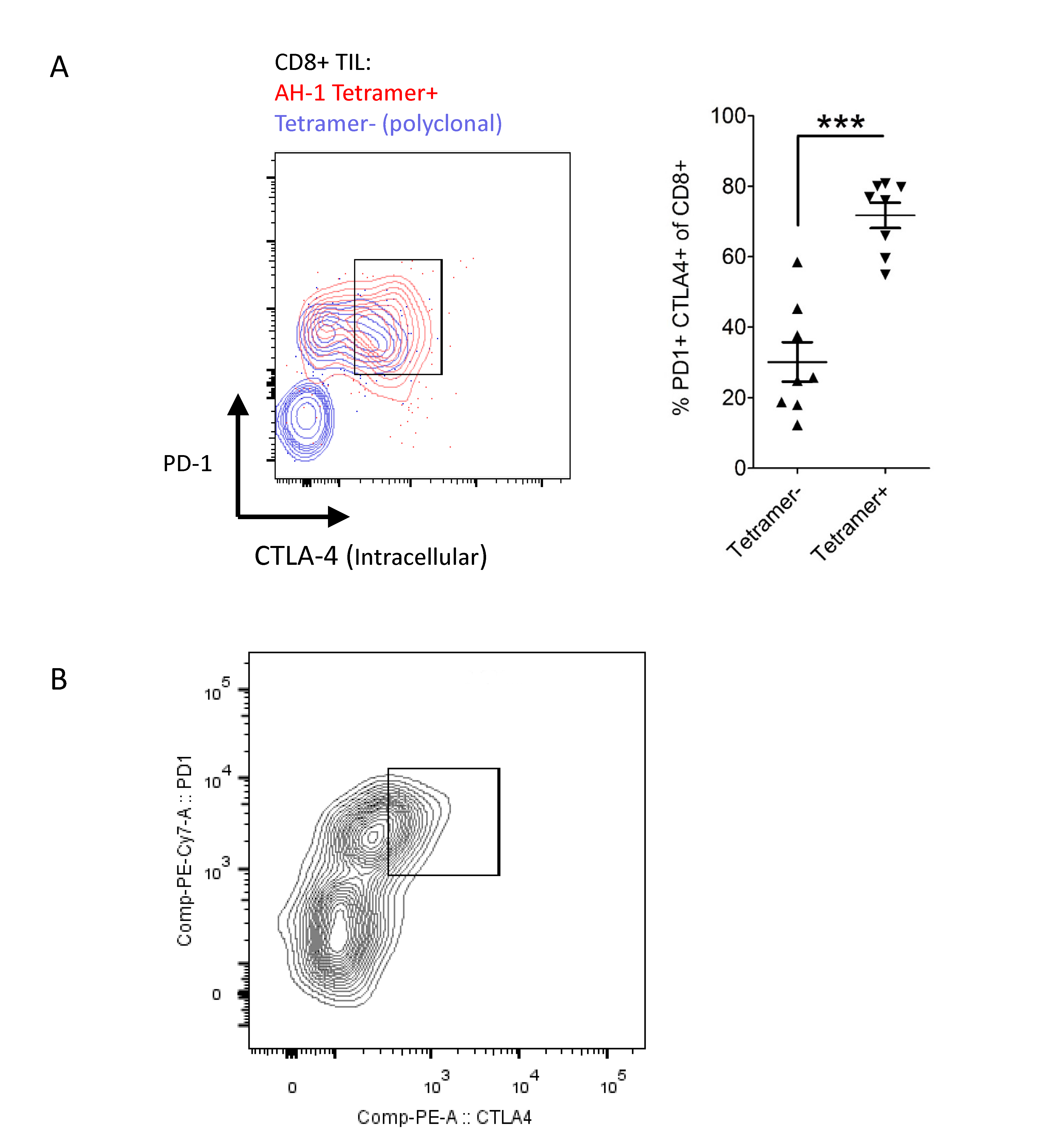

Supplement: S2 Fig — A. TIL from CT26 tumor-bearing mice were harvested 15 days after tumor implantation. On left, frequencies of PD-1/CTLA-4 co-expression in each population are shown. *** p<0.001. On right, representative overlaid plot of PD-1/CTLA-4 (ICS) co-expression in AH-1 tetramer+ CD8+ TIL (red) and AH-1 tetramer- CD8+ TIL (polyclonal, blue). B. PD-1 and CTLA-4 co-expression on polyclonal CD8+ T cells harvested from MC38 tumors 15 days after implantation. (TIFF) [file pone.0161779.s002.tiff]

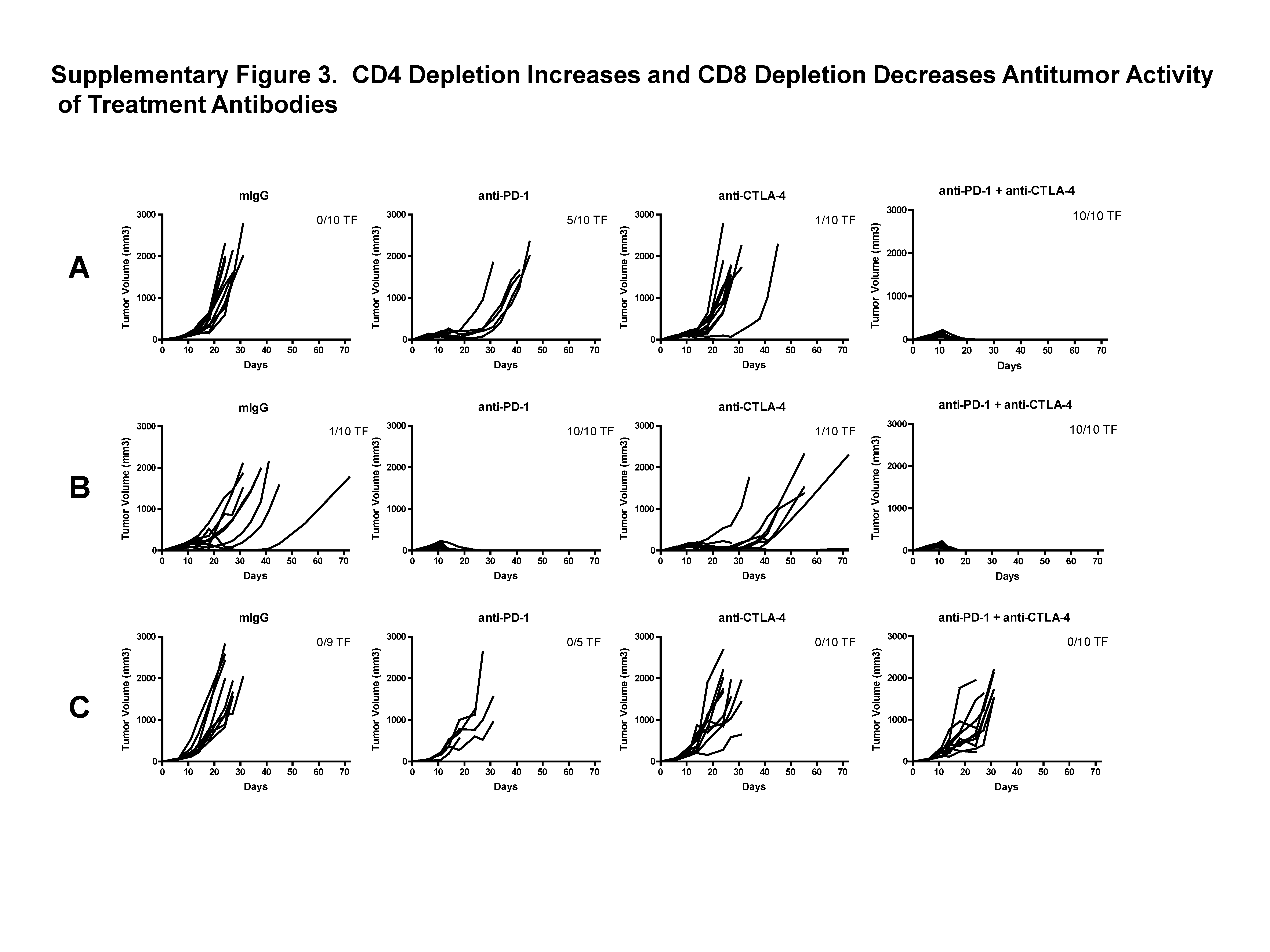

Supplement: S3 Fig — A. C57BL/6 mice were with injected 2×10^6^ MC38 cells and treated on days 7, 10, and 13 with 400 μg of isotype control or single agent, or 200 μg of each agent for the combination therapy. Anti-PD-1 vs control p = 0.0013; anti-PD-1 and anti-CTLA-4 vs control p<0.0001. B. Treatment of mice as in A with the addition of 500 μg of depleting CD4 mAb (GK1.5 BioXCell) i.p. on day 7. Anti-PD-1 vs control p = 0.0001; anti-PD-1 and anti-CTLA-4 vs control p<0.0001. C. Treatment of mice as in A with the addition of 500 μg of depleting CD8 mAb (53.6.72 BioXCell) i.p. on day 7. Anti-PD-1 and anti-CTLA-4 vs control p = 0.0149. The number of tumor-free (TF) mice per group is shown. FACS analysis of group B and C blood samples confirmed >90% depletion of CD4+ and CD8+ T cells four days after administration of depleting antibodies. (TIFF) [file pone.0161779.s003.tiff]

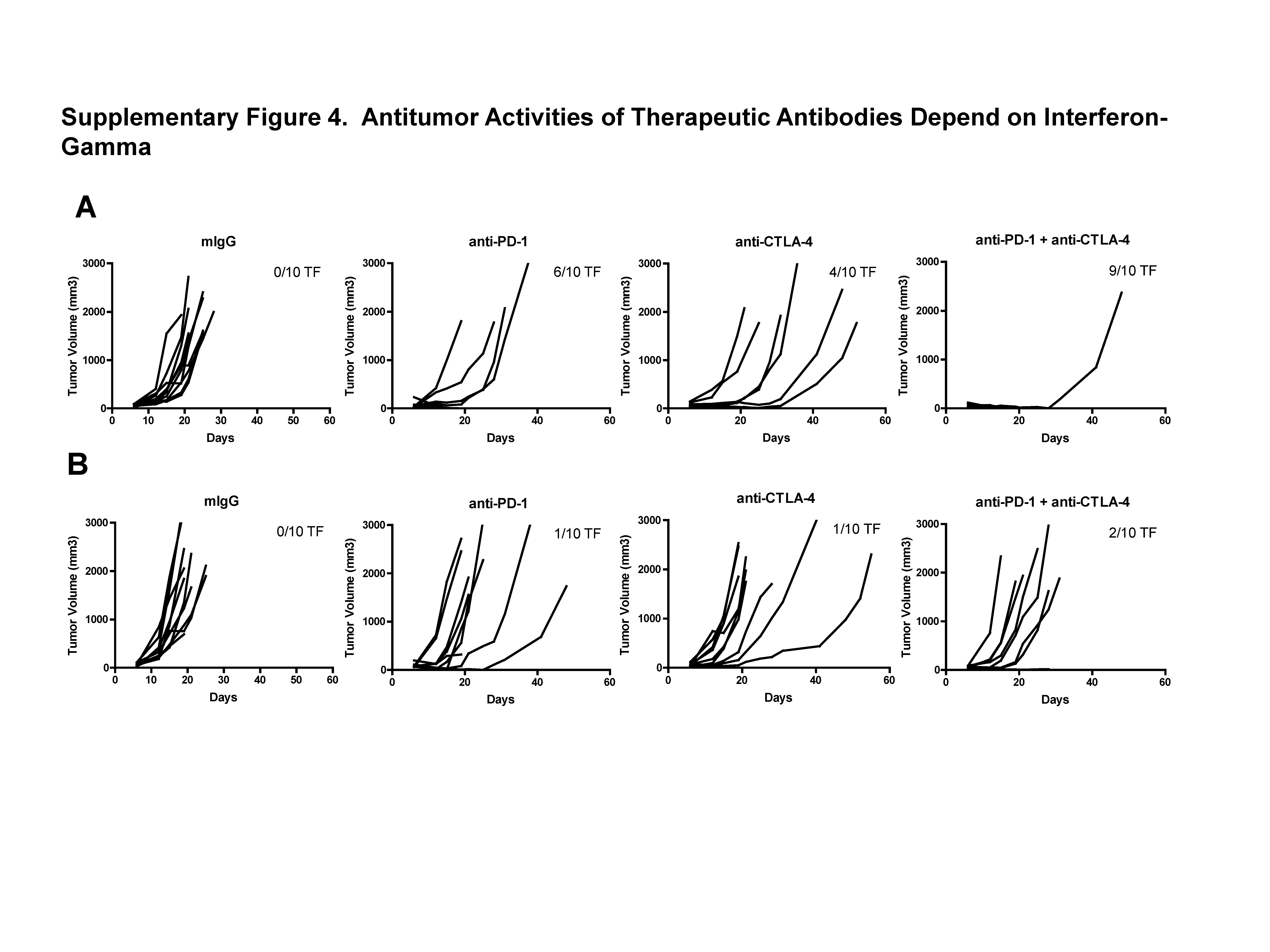

Supplement: S4 Fig — Wild-type (A) or B6.129S7-Ifngtm1Ts/J (B) C57BL/6 mice were injected with 2×10^6^ MC38 tumor cells and treated with 400 μg of isotype IgG1 or 200 μg of isotype with 200 μg of anti-PD-1 or anti-CTLA-4, or 200 μg each of anti-PD-1 and anti-CTLA-4 for the combination therapy on days 0, 4, and 7. The number of tumor-free (TF) mice per group is shown. A. anti-PD-1 vs control p = 0.0082; anti-CTLA-4 vs control p = 0.0464; anti-PD-1 and anti-CTLA-4 vs control p<0.0001. B. anti-PD-1 and anti-CTLA-4 vs control p = 0.0264. (TIFF) [file pone.0161779.s004.tiff]

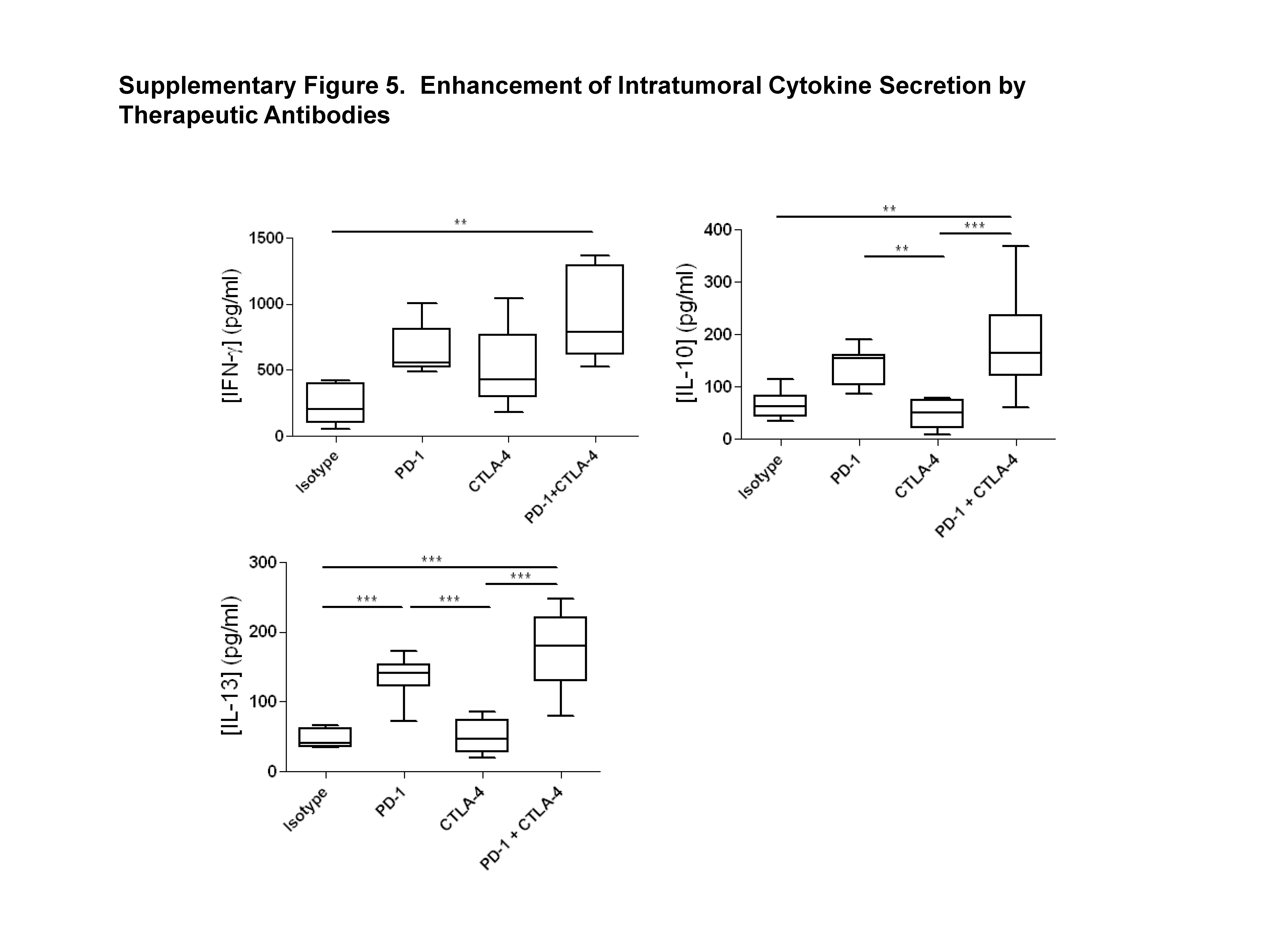

Supplement: S5 Fig — C57BL/6 mice were injected with 2×10^6^ MC38 cells and treated on days 7, 10, and 13 with 200 μg of isotype control or single agents, or 200 μg of each agent for the combination therapy. On day 15 post implantation, tumors were harvested, manually dissociated into single-cell suspensions, and levels of intratumoral cytokines were assessed via bead-based cytokine arrays (FlowCytomix, eBioscience). Results are shown for IFN-γ, IL-10, and IL-13. (TIFF) [file pone.0161779.s005.tiff]

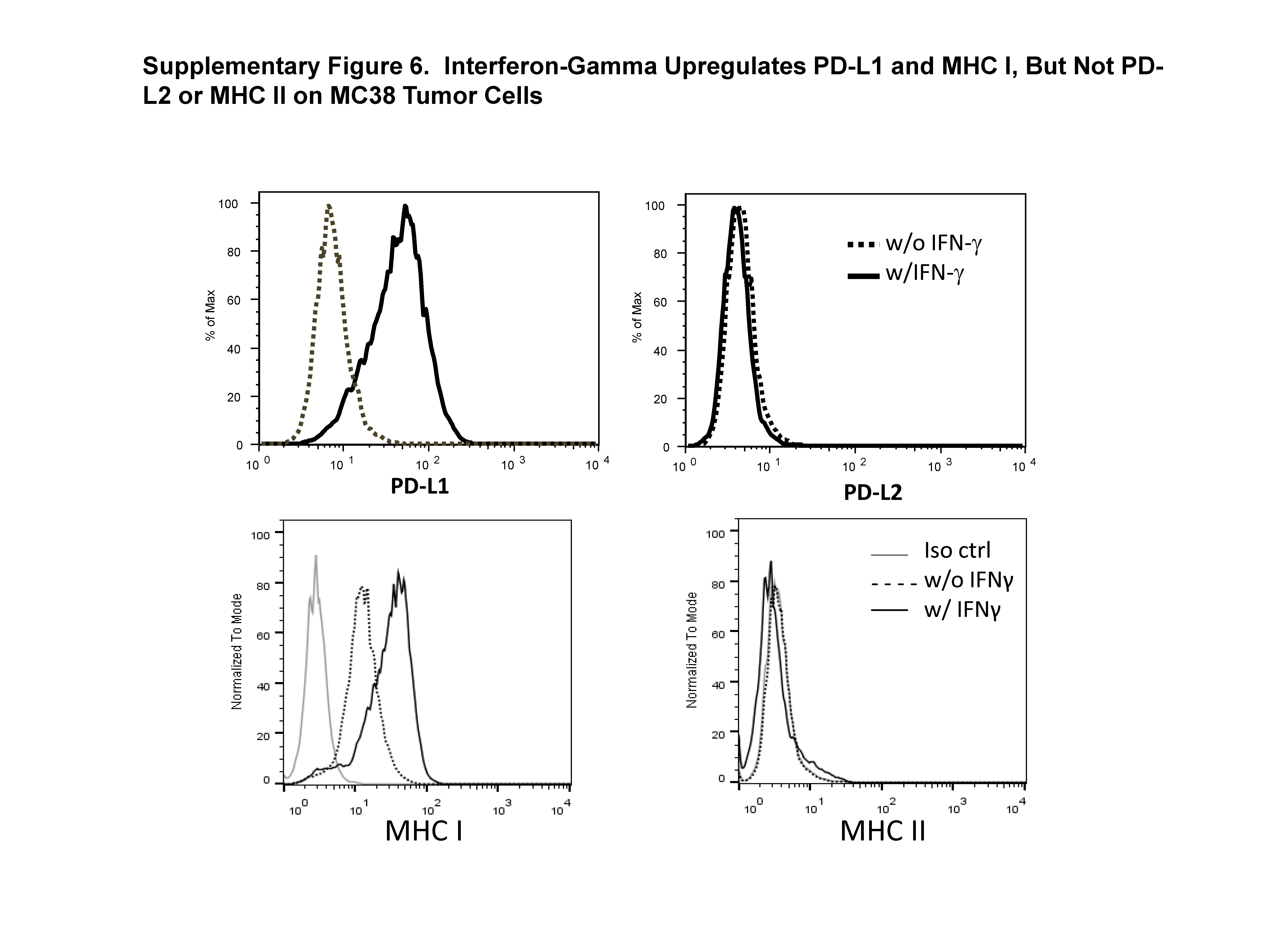

Supplement: S6 Fig — MC38 cells were cultured in the presence of recombinant murine IFN-γ (R&D Systems) at 5 ng/mL for 24 hours and analyzed by FACS for expression of mouse PD-L1 (eBioscience, MIH5), mouse PD-L2 (eBioscience, 122), mouse MHC I (H-2Db; eBioscience 28-14-8), or mouse MHC II (I-A/I-E; eBioscience M5/114.15.2). (TIFF) [file pone.0161779.s006.tiff]

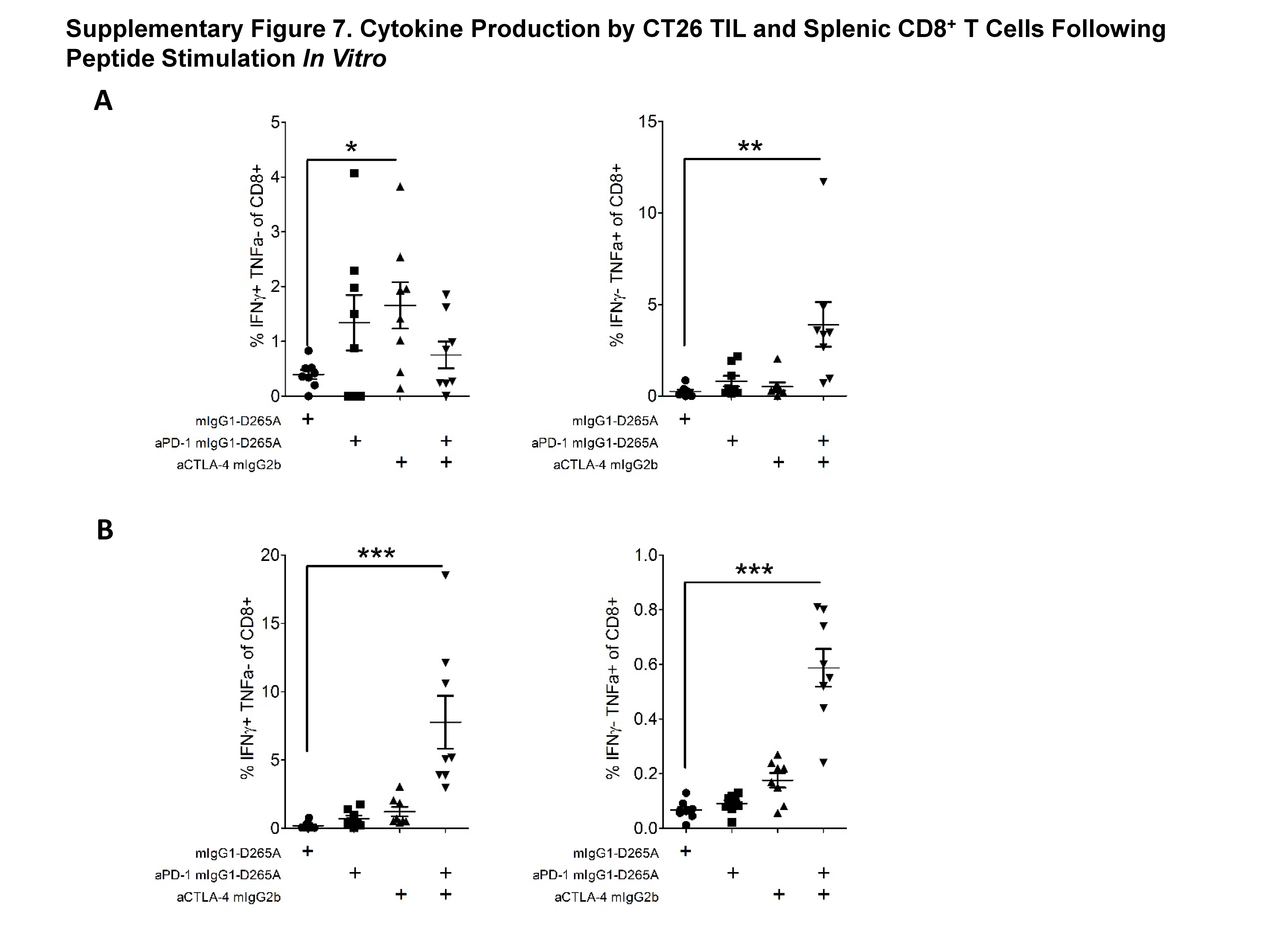

Supplement: S7 Fig — CT26 colon tumor cells (1x10^6^) were implanted subcutaneously into BALB/c mice, which were then treated and analyzed as described in Fig 3. A. Frequencies of IFN-γ+ TNF-α- and IFN-γ- TNF-α+ cells of total tumor-infiltrating CD8+ T cells following in vitro AH1 peptide stimulation. B. Frequencies of IFN-γ+ TNF-α- and IFN-γ- TNF-α+ cells of total splenic CD8+ T cells following in vitro AH1 peptide stimulation. * p<0.05; ** p<0.01; *** p<0.001. (TIFF) [file pone.0161779.s007.tiff]
